# Relationship between socio-linguistic factors and symptoms of depression, anxiety and psychosis in the general population

**DOI:** 10.3389/fpsyg.2026.1690424

**Published:** 2026-02-20

**Authors:** Naiara Ozamiz-Etxebarria, Leire Erkoreka, Simona Mancini

**Affiliations:** 1 University of the Basque Country UPV-EHU, Leioa, Spain; 2 Galdakao-Usansolo University Hospital, Osakidetza Basque Health Service, Galdakao, Spain; 3 BioBizkaia Health Research Institute, Barakaldo, Spain; 4 CIBERSAM ISCII, Madrid, Spain; 5 Basque Center on Cognition, Brain and Language, Donostia-San Sebastian, Spain; 6 Ikerbasque, Basque Foundation for Science, Bilbao, Spain

**Keywords:** bilingual, education, language proficiency, mental health, sociocultural context

## Abstract

**Introduction:**

Over the last decade, and especially since the COVID-19 pandemic, psychological distress has increased significantly in the general population, with a particularly strong impact on young people. This situation has intensified interest in identifying risk and protective factors associated with mental health. Within this framework, the present study aimed to analyze the relationship between sociodemographic and linguistic variables and different indicators of mental health in a bilingual population from the Basque Country.

**Methods:**

A total of 521 bilingual individuals (Basque/Spanish) from the general population participated in the study. Participants completed validated questionnaires assessing anxiety (GAD-7), depression (PHQ-9), and prodromal psychotic symptoms (PQ-B), along with a sociodemographic questionnaire and a measure of linguistic competence. Univariate analyses and linear regression models were conducted to examine associations between mental health indicators and sociodemographic variables (age, gender, educational level, employment status, marital status, and offspring), as well as linguistic variables (self-perceived proficiency in local languages and foreign language certification).

**Results:**

The results indicate that higher educational attainment is consistently associated with lower levels of depressive symptoms and prodromal psychotic symptoms. Additionally, better self-perceived competence in the usual languages of communication (Basque and Spanish) is related to lower scores on prodromal psychotic symptoms. Having children is associated with lower levels of depressive symptoms. In contrast, variables such as gender, marital status, and employment status did not show significant associations with mental health indicators in this predominantly young sample.

**Discussion:**

Overall, these findings highlight the relevance of educational level and sociolinguistic context as important factors in mental health. They underscore the need to incorporate educational and linguistic dimensions into the design of prevention and intervention strategies, particularly those targeting young populations in bilingual and multilingual contexts.

## Introduction

Worldwide, there has been a marked increase in psychological distress in recent years (Keyes and Platt, 2024). While this upturn had already been recorded prior to the COVID-19 pandemic, the health crisis significantly exacerbated mental health problems in various populations (Brito et al., 2022; Daniali et al., 2023; Zhang et al., 2022). The WHO (World Health Organization, 2022a) reported a 25% rise in the global prevalence of depression and anxiety disorders during the first year of the pandemic. This increase is due to factors such as occupational stress, lack of support networks and economic uncertainty, all of which may increase psychological vulnerability. Studies in various parts of the world have found the younger population to be most affected, with a significant rise in the incidence of mood and anxiety disorders (Marelli et al., 2021; Qiu et al., 2020; Solomou and Constantinidou, 2020). It has been suggested that this vulnerability in young people may be related to factors such as employment insecurity, overuse of social media and lack of coping skills (Ozamiz-Etxebarria et al., 2023). In many regions, structural factors, such as lack of access to mental health services and the stigma associated with mental disorders, have also contributed to the mental health crisis (World Health Organization, 2022b).

Gender-related differences in mental illness have also been extensively documented, in studies showing that women have higher rates of anxiety, depression and eating disorders, while men have a higher prevalence of addiction, attention deficit hyperactivity disorder, psychosis, schizophrenia and autism spectrum disorders. These differences cannot be explained by biological factors alone; rather, they appear to be profoundly influenced by gender inequalities in society. Women, for example, experience higher levels of stress due to the burden of unpaid work, job insecurity and gender-based violence, which all contribute to their greater emotional vulnerability (Hutten et al., 2021). In addition, there is a real issue with the medicalization of female mental health, with a greater tendency to diagnose depression and anxiety among women and to prescribe psychotropic drugs more frequently than among men, even when they present with similar symptoms (Artazcoz et al., 2018).

Several studies have also shown a relationship between employment and mental health. Different dimensions of precarious employment such as job insecurity and seasonality are associated with mental health problems (Rönnblad et al., 2019; Utzet et al., 2020). In addition, unemployment is associated with impaired mental health (Paul and Moser, 2009), with men the most affected (Murphy and Athanasou, 1999; Virgolino et al., 2022).

Marital status has also been observed to play an important role in mental health (Christensen et al., 2022). One study found that married people scored higher for psychological well-being due to the social support and economic resources associated with marriage (Siva Kumar et al., 2019). Other studies found an association between being single and indicators of mental illness (Siva Kumar et al., 2019). Marital status (in this case, being married) would therefore appear to be a protective factor in mental health (Rohrer et al., 2005). Nonetheless, a separate study found that the relationship between marital status and depression varies by age and gender, with a lower risk among single, widowed and separated women and the risk increasing with age among single men (Bulloch et al., 2017).

With regard to educational level, research has shown that a higher level of education may be associated with a lower rate of mental illness (Li et al., 2023; Xu and Chen, 2024). Thus, the prevalence of mental disorders is greater among those with lower educational levels, suggesting that higher levels of education are associated with better mental health outcomes. In addition, one study observed that people with a high level of education generally utilize mental health services more and rely less exclusively on medication; this could be interpreted as a more comprehensive management of their mental health (Halme et al., 2023).

Finally, learning new languages could be identified as a possible protective factor. Language learning has been described as being a therapeutic activity that can improve mental health (de Diego Alonso and Isiegas, 2007). Other findings show that anxiety is inversely related to the number of languages learned (Thompson and Lee, 2013) and that perceived language proficiency is positively related to several aspects of psychological well-being (Luo et al., 2019), although this association has not been explored to any great degree. Moreover, mental health issues can make it difficult to learn new languages (Wu, 2024). In any case, studies in this area are scarce and there is much yet to be explored.

Considering the relevance of these variables, this study seeks to analyze the relationship between sociodemographic and linguistic factors and mental health, providing empirical evidence on the influence of gender, marital status, cohabitation, offspring, employment status, educational level, and knowledge and self-perception of language proficiency on anxiety, depression and psychotic symptoms.

## Materials and methods

### Participants and procedures

The study sample consisted of 521 bilingual (Basque/Spanish) adults from the general population who participated in a broader research project (Erkoreka et al., 2025). Among them, 383 participants (73.5%) identified as female, 131 (25.1%) as male, and 7 (1.3%) as non-binary. The mean age of the sample was 26.58 years (SD = 7.67), most participants were single (68.9%), and the majority were working or studying at the time of data collection (83.7%). Participants lived in households with a mean of 3.37 cohabitants (SD = 1.18). Educational attainment was generally high, with 73.7% having completed university studies, 25.5% high school or vocational training, and less than 1% primary education. A minority of participants (11.9%) reported having offspring. From a linguistic perspective, participants reported high self-perceived proficiency in Basque and Spanish (mean = 73.32, SD = 5.86), and 67% held an official qualification in at least one foreign language (Table 1).

**Table 1:** Description of the sample.

Variables	Variables	Mean ± SD			
		Total (*N* = 521)	Male (*N* = 131)	Female (*N* = 383)	Non-binary (*N* = 7)
Age*		26.58 ± 7.67	25.55 ± 7.6	25.98 ± 7.6	22.29 ± 1.1
Number of cohabitants		3.37 ± 1.18	3.25 ± 1.13	3.40 ± 1.20	3.71 ± 1.11
Proficiency in local languages		73.32 ± 5.86	72.36 ± 6.66	73.68 ± 5.55	71.57 ± 4.92
GAD-7		6.48 ± 3.84	5.89 ± 3.95	6.67 ± 3.77	7.00 ± 4.47
PHQ-9		7.07 ± 5.10	5.57 ± 4.86	7.23 ± 5.17	7.86 ± 5.92
PQB		3.13 ± 3.35	2.88 ± 3.32	3.20 ± 3.36	4.06 ± 1.52
		N(%)			
Offspring (yes)		62(11.9)	20 (15.3)	42 (11.0)	0 (0)
Marital status	Single	359 (68.9)	88 (67.2)	266 (69.6)	5(71.4)
	Married/partner	106 (20.3)	29 (22.1)	77 (20.2)	0 (0)
	Divorced	3(0.6)	1 (0.8)	2 (0.5)	0 (0)
	Others	53 (10.2)	13 (9.9)	37 (9.7)	2 (28.6)
Occupation	Not working	85 (16.3)	15 (11.5)	69 (18)	1 (14.3)
	Working/Studying	436 (83.7)	116 (88.5)	314 (82)	6 (85.7)
Education	Primary	4(0.8)	1 (0.8)	3 (0.8)	0
	High school/Vocational	133 (25.5)	32 (24.4)	99 (25.8)	2 (28.6)
	University	384 (73.7)	98 (74.8)	281 (73.4)	5 (71.4)
Foreign language qualification (yes)		349 (67)	90(68.7)	256 (66.8)	3(42.9)

GAD-7, Generalized Anxiety Disorder Scale-7; PHQ-9, Patient Health Questionnaire-9; PQB, Prodromal Questionnaire-Brief Version. *Statistically significant results were observed between males’ and females’ scores, with no differences regarding non-binary people.

Participants were eligible if they were 18 years of age or older, resided in the Basque Country, were bilingual in Basque and Spanish, and provided informed consent. Individuals were excluded if they did not complete the questionnaires, provided inconsistent responses, or did not meet the bilingualism criteria.

Recruitment was carried out between November 2019 and January 2021 through convenience sampling, mainly via university mailing lists, the BCBL volunteer database, and word of mouth. Those interested accessed the online forms via Google Forms, first agreeing to the informed consent form and then completing the sociodemographic questionnaire and the clinical and linguistic assessments. All measures were completed in a single session, with an estimated duration of 20–30 min, and data were collected anonymously.

The study employed a cross-sectional, observational, and correlational design, which was considered appropriate for examining associations between sociodemographic and linguistic variables and mental health indicators in a non-clinical population. Although causal inferences cannot be made, this design allows the identification of potential risk and protective factors relevant for prevention and intervention strategies.

### Measures and instruments

Generalized Anxiety Disorder Scale-7 (GAD-7, Patel et al., 2019): This is a self-administered questionnaire designed to assess the presence and intensity of generalized anxiety disorder (GAD) symptoms over the previous 2 weeks, following the diagnostic criteria established in the DSM-IV-TR. It includes 7 items, each rated on a 4-point Likert scale. A threshold score of 10 or higher has been suggested for detecting probable cases of GAD. Scores of 5, 10, and 15 are commonly used to categorize anxiety as mild, moderate, and severe, respectively. The measure has demonstrated strong psychometric properties across diverse languages and populations (Lee et al., 2022; Vrublevska et al., 2022), including excellent internal consistency, with Cronbach’s alpha ranging from 0.85 to 0.95.

Patient Health Questionnaire-9 (PHQ-9, Kroenke et al., 2001): This self-administered tool comprises 9 items, each corresponding to one of the DSM-IV criteria for depressive disorders, rated on a 4-point Likert scale. An additional item assesses the degree of functional impairment caused by the symptoms. A total score of 10 or above is typically used as a cut-off to suggest the presence of a potential depressive disorder. The instrument has been validated across multiple languages and cultural settings (Fonseca-Pedrero et al., 2018; Gómez-Gómez et al., 2023; Jomli et al., 2020; Lamela et al., 2020), consistently demonstrating solid internal reliability, with Cronbach’s alpha coefficients ranging from 0.79 to 0.89.

Prodromal Questionnaire-Brief Version (PQ-B, Loewy et al., 2011): This screening tool for psychosis risk is a self-report questionnaire comprising 21 dichotomous (yes/no) items and is based on symptom frequency. The PQ-B is a shortened version derived from the original 92-item Prodromal Questionnaire (Loewy et al., 2011). When a respondent answers “yes” to an item, a follow-up question is presented to assess both the frequency and level of distress or functional impact associated with the experience, on a 5-point Likert-type scale. Various studies have proposed different cut-off scores for the frequency and distress subscales, depending on the population under study (Kaligis et al., 2018; Okewole et al., 2015; Pelizza et al., 2018). In the case of the Spanish population, validation studies have recommended a threshold score of 9 or over on the frequency scale (Fonseca-Pedrero et al., 2018) and 29 or over on the distress scale (Fonseca-Pedrero et al., 2016). These validations also report strong internal consistency (Cronbach’s alpha between 0.83 and 0.89) and support a unidimensional factor structure for the instrument.

BCBL language competence questionnaire: This questionnaire was developed by the Basque Center on Cognition, Brain and Language as a tool for making a comprehensive linguistic assessment of an individual. Among other variables, it collects information on the languages spoken by the person, their exposure, self-estimated proficiency in each language, and whether they have official qualifications in any of the languages they know. This study considered self-estimated proficiency in local languages (Basque and Spanish) as a sum of the two scores ranging from 40 to 80 and whether the individual has any official qualification in a foreign language (Erkoreka et al., 2025).

### Data analysis

We first performed univariate analyses to study the associations between sociodemographic and linguistic variables and the scores on the three scales. We used Pearson’s correlation to study the relationship between quantitative variables and Student’s t test for qualitative variables. Secondly, we created three linear regression models, in which the dependent variable was the score on the scale studied (GAD7, PHQ-9 and PQ-B) and the independent variables were those sociodemographic and linguistic variables which had shown a significant (*p* < 0.1) association in the univariate analyses. We used IBM SPSS Statistics 29.0 for the statistical analyses.

## Results

Table 2 shows the results obtained in the 3 linear regression models. With regard to symptoms of anxiety, the only variables to show an association in the univariate analyses were sociodemographic (offspring, age and educational level), but none of these survived when included jointly in the regression model. A trend can be seen in education level, suggesting that the higher the education level, the less the anxiety experienced, but without attaining the established level of statistical significance.

**Table 2:** Regression models.

Models	Variables	β	t	*p*	Adjusted R^2^
GAD-7 (1)	Constant		9.977	<0.001	0.014
	Education	−0.077	−1.727	0.085	
	Age	−0.052	−0.958	0.339	
	Offspring	−0.065	−1.217	0.224	
PHQ-9 (1)	Constant		5.405	<0.001	0.048
	Education	−0.150	−3.355	<0.001	
	Age	−0.044	−0.805	0.421	
	Offspring	−0.095	−1.813	0.070	
	Proficiency	−0.065	−1.463	0.144	
	Occupation	−0.043	−0.990	0.323	
	Foreign qual.	−0.056	−1.244	−214	
PHQ-9 (2)	Constant		12.530	<0.001	0.045
	Education	−0.176	−4.088	<0.001	
	Offspring	−0.120	−2.795	0.005	
PQB (1)	Constant		6.196	<0.001	0.074
	Education	−0.175	−3.983	<0.001	
	Offspring	0.018	0.347	0.729	
	Proficiency	−0.105	−2.406	0.016	
	Foreign qual.	−0.065	−1.482	0.139	
	Age	−0.145	−2.718	0.007	

GAD-7, Generalized Anxiety Disorder Scale-7; PHQ-9, Patient Health Questionnaire-9; PQB, Prodromal Questionnaire-Brief Version.

With regard to clinical depression, multiple sociodemographic variables (offspring, job, educational level, age) and linguistic variables (foreign language certification, proficiency in local languages) showed a relationship with the PHQ-9 score in univariate analyses. When, in a preliminary step, all the variables are incorporated into the regression model, it is evident that only the education level continues to show a significant association, and a trend can be observed in having offspring. In the second step, we removed the variables for which the association did not endure, showing that educational level and offspring were significantly related to depressive symptomatology, such that a higher educational level and having children were associated with lower scores for depression. These two variables explain 4.5% of the variability of the PHQ-9 score.

Finally, with regard to the study of psychotic symptoms, there was again found to be a relationship between both sociodemographic and linguistic variables (age, level of education, offspring, foreign language qualification and proficiency) and the PQ-B score. Incorporating all of these into the regression model, we observed that the significant effect of educational level, proficiency and age survives the control for multiple variables, with the model explaining 7.4% of the variability in the PQ-B score. Higher educational level, greater language proficiency in local languages and older age are associated with lower scores for psychotic symptomatology.

## Discussion

This study analyzed the relationship between several sociolinguistic factors and mental health, providing evidence of their impact on the symptoms studied.

To begin with, the results obtained in the univariate analyses indicate that gender is not significantly associated with the mental health indicators assessed by PHQ-9, GAD-7 and PQ-B. These findings contrast with those of previous studies that have identified gender differences (Hutten et al., 2021; Eugene et al., 2025) in the prevalence of mental illness. One possible explanation is that because they measure general symptoms of mental health, the tools used in this study may not capture gender-specific nuances, such as different ways of expressing or manifesting psychological distress, which might limit the detection of differences among the population evaluated.

Occupation and marital status have not been found to influence symptomatology either. This finding contrasts with those of previous studies (Begum, 2025; Compernolle and Zheng, 2025; Murphy and Athanasou, 1999; Paul and Moser, 2009; Virgolino et al., 2022). However, with regard to marital status, it may be due to the fact that the mean age of our population was quite young (26.58 years), and as already noted in previous studies (Bulloch et al., 2017; Solomou and Constantinidou, 2020), the influence of this factor appears to increase with age. As for the influence of occupation, one possible explanation might be that the young people in this sample have jobs with more favorable or less demanding conditions, which have a subsequently reduced impact on their well-being.

With regard to age, an inverse association with all indicators of the symptomatology was observed in the univariate analyses, but this relationship remained significant only in the model adjusted for psychotic symptomatology. This finding is consistent with recent studies that indicate an increased vulnerability to subclinical psychotic experiences among the younger population (Fonseca-Pedrero et al., 2009; Lemos-Giráldez et al., 2011; Ozdemir et al., 2025).

Of all the items studied, educational level appears to be the most determinant for the three spectra of symptoms. In the post-hoc study carried out after the univariate analysis, we found that the difference was mainly between the high school/VT group and university students for all three questionnaires, with a U-shaped pattern, although the regression models indicate that the higher the level of studies, the lower the rate of symptoms observed. Several studies have shown that a higher educational level is associated with better mental health (Bracke et al., 2014; Gariépy et al., 2022; Kondirolli and Sunder, 2022; Sesti et al., 2022; Sperandei et al., 2023; Xu and Chen, 2024). This suggests that education may act as a protective factor against mental disorders by promoting the development of personal, professional and social resources that contribute to psychological well-being.

In our study, proficiency in an additional third or fourth language was not found to be significantly related to reduced symptomatology, as described in previous research highlighting the cognitive and emotional benefits of language learning (de Diego Alonso and Isiegas, 2007; Thompson and Lee, 2013). However, there was an inverse relationship between self-perception of proficiency in local languages (Basque and Spanish) and PQ-B, indicating that the better the perception of proficiency in their habitual languages of communication, the lower the rate of psychotic symptomatology. This would be an important finding of this study, reinforcing and expanding upon aspects described in other previous studies that found that language proficiency was positively related to several aspects of psychological well-being and mental health (Luo et al., 2019; Montemitro et al., 2021).

As a final sociodemographic variable of interest, we found that having children appears to have a protective effect against symptoms of depression. This is especially relevant given that most previous studies have focused on analyzing how depression influences the probability of having children, generally identifying a negative association between the two (Golovina et al., 2023; Kailaheimo-Lönnqvist et al., 2024). Our study therefore offers a complementary perspective, and although the cross-sectional design does not allow us to establish causality, it does point to a novel and interesting association between parenthood and greater psychological well-being.

This study has some limitations that must be taken into account. To begin with, participation in this study is higher among women and the number of non-binary individuals is negligible. Nonetheless, it is important to note that there are no significant differences among the three groups across the depressive, anxious and psychotic symptomatology, as addressed by these widely used instruments. The fact that there are more women than men in the samples is quite common in many studies (Ganguli et al., 1998), and even more so in studies on psychology, education, and mental health. The topics of many mental health studies tend to arouse greater interest or identification among women, which increases their participation (Proyer and Häusler, 2007). Cultural and social factors also play a role, as women may feel more comfortable or less stigmatized when talking about emotions and psychological well-being (Deng et al., 2016). Finally, recruitment or sampling methods through universities, associations or social networks may favor the recruitment of women, as these are areas where they are more present. There are no official figures on the prevalence of people who identify as non-binary in our setting, so we cannot venture to determine whether they are over- or underrepresented in our sample.

Another limitation of this study is the age range of the participants. The sample included young people and young adults, mainly ranging from 18 to 33 years of age. Therefore, in the context of this study, participants who fall into the category of ‘young adults’ according to the WHO classification (2023) are included. For this reason, the conclusions of this study must be limited to that age range.

It is also important to mention that despite most data was collected before the COVID-19 pandemic, the recruitment period was extended until 2021, and we know that young people’s mental health has worsened during the pandemic (Idoiaga et al., 2022; Ozamiz-Etxebarria et al., 2023). Therefore, and although dispersion of the scores on the PHQ-9 and GAD-7, which assess symptoms that may have increased due to the pandemic, is within the usual range for these instruments, we cannot rule out that they may be oversized in the months following the pandemic.

Finally, the way in which the study has been disseminated, mainly through mailing lists, may favor self-selection bias (those who are more engaged, have more free time, or feel more strongly about a topic may be overrepresented). The use of self-administered questionnaires may also be associated with biases such as social desirability, recall bias, or extreme response bias. However, we must bear in mind that the tools used, mainly the PHQ-9 and the GAD-7, are strongly supported and are, in fact, the main assessment instruments used in mental health research.

## Conclusion

In conclusion, this study provides evidence that educational level may play a central role in mental health, acting as a protective factor against symptoms of depression, anxiety, and psychosis.

Higher education appears to contribute to psychological well-being, likely through the development of personal, social, and professional resources.

Additionally, having offspring was associated with lower depressive symptomatology, suggesting that parenthood may offer emotional benefits, although this finding should be interpreted cautiously due to the cross-sectional nature of the study. In terms of psychotic symptoms, older age and higher self-perceived proficiency in local languages (Basque and Spanish) were linked to lower symptom rates, highlighting a novel potential protective role of language proficiency in habitual communication.

No significant associations were found with gender, marital status, or employment, which may be related to the predominantly young, educated, and university-based nature of the sample.

Overall, these results underscore the importance of focusing on the mental health of young adults and suggest that interventions aiming to improve psychological well-being could benefit from promoting access to higher education, supporting parenthood, and enhancing language proficiency. Future research should examine these associations longitudinally, explore underlying mechanisms, and investigate whether these findings generalize to broader and more diverse populations.

## Data Availability

The data that support the findings of this study are available from the corresponding author upon reasonable request.
